# Miscellaneous Surgical Essays

**Published:** 1781-07

**Authors:** 


II. *Vermischte chirurgische schriften, &c.* i. e. *Miscellaneous surgical Essays, published by* John Leberecht Schmucker, *First Surgeon General to the army, and Director of all the military hospitals in Prussia.* Vol. II. 8vo. Berlin. 302 pages, with a copper-plate.

OF the first volume of this collection we gave an ample account in a former part of our Journal. The essays contained in this second volume are as follows:

I. *On amaurosis, by* Mr. Schmucker.—The author sets out with observing, that in this disease the pupil of the eye gradually loses its splendor, and acquires a pale colour, so that a skilful practitioner is able, even at a distance, to judge of the presence of blindness. He remarks that, in general, patients labouring under an imperfect amaurosis, see worse in clear day-light than in obscure places. In many cases the disease comes on suddenly, and in such, it seems, he has commonly been more successful than where the complaint was slow in its progress. In cases of incipient amaurosis, he has sometimes observed that when both eyes were open, the patients saw objects double.

He observes that the curative indications in this disease will be as various as its causes. The most most frequent of these he supposes to be an increased determination to the head from a suppression of the hæmorrhoids or catamenia, in either of which cases a return of the flux is to be promoted by applying leeches to the anus or labia pudendi. Mr. Schmucker recommends at the same time copious venæsection, the application of leeches to the throat and temples, and a perpetual blister to the neck.

In cases where the energy of the optic nerve seemed to be impaired, he has experienced excellent effects from nauseating doses of emetic tartar given twice a day for several weeks.

In two cases where the disease was accompanied by an obstruction of the frontal sinuses, and a preternatural dryness of the nostrils, he succeeded in the cure by means of snuff, which by promoting a discharge from the nose gradually removed the complaint.

The author observes that in fevers, small pox, measles, gout, &c. this disorder is often the effect of metastasis, and he gives an instance of a patient who, upon being troubled with a gouty pain in his heel, applied cold water to the part affected. The pain immediately ceased, but the next day he became blind. He likewise speaks of another patient who was affected in a similar manner by repelling the rheumatifm from his fhoulder by cold applications. In this laft cafe Mr. Schmucker fucceeded in the cure by the following mode of treatment. He began by applying a blifter between the fhoulders, and prefcribing an emetic, which was repeated the day following. In three days the patient began to recover his fight, and by means of the naufeating courfe juft now mentioned, together with the ufe of millepedes infufed in Rhenifh wine, and of an embrocation to the temples, the moft active ingredients of which are fpir. lavend. c. & fpir. fal. ammoniac. the cure was compleated in a few weeks. A fimilar method, we are told, proved equally efficacious in a cafe of amaurofis brought on by the meafles.

Mr. Schmucker remarks that the venereal amaurofis requires the ufe of mercury, but that in all other cafes this remedy is hurtful. In feveral patients he has tried the flowers of arnica, and in others electricity, but without experiencing the leaft good effect from either of thofe remedies. It ought to be obferved, however, that the new method of adminiftering electricity lately adopted in this country was unknown to our author.

II. *Remarks on tranflations of milk from the breafts, by* the fame.—Two cafes of abfcefs are here related which the author, perhaps without fufficient reafon, afcribes to a tranflation of milk from the breafts.

III. *A cafe of hernia, by* the fame.—The moft remarkable circumftance in this cafe, previous to the operation, was a hard lump which formed a part of the hernia, and could not be reduced. On making an incifion into the hernial fac it was found to contain a confiderable quantity of water, and the lump juft now mentioned was a portion of the omentum adhering to the fac. The whole of this fubftance was fuccefsfully removed, but not without a fecond operation, as the patient's lofs of ftrength did not allow it to be completely extirpated at once.

Mr. Schmucker obferves that the fpermatic chord and tefticle were found lying before the hernial fac, fo that they might have been eafily wounded by a hafty or inexperienced operator.

IV. *Of a dangerous wound of the head, by* the fame.—In this cafe the trepan was repeatedly applied on account of a wound on the left parietal bone. On the nineteenth day after the operation the face fwelled, and the patient was feized with a locked jaw. Thefe alarming fymptoms, however, ever, were removed by a diarrhœa which came on the 25th day, and continued for fome time.

V. *Of the amputation of fingers and toes, by* Mr. Block.—Several cafes are defcribed in this paper. The firft relates to the amputation of a fore-finger of immenfe bulk. It had been for a long time painful and ulcerated, and when taken off was found to weigh twelve ounces and a half. Externally it was of a cartilaginous confiftence, but within it was merely gelatinous, affording no appearance of mufcles or tendons. Three cafes are fpoken of, of fingers cut through by a broad fword, all of which our author contrived to cure by what in furgery is termed the firft intention.

Mr. Block fpeaks of a woman who was repeatedly delivered of children with fix fingers and toes, though neither fhe or her hufband had more than five. In fome of thefe inftances the fingers or toes being imperfect, and without a bone, our author thought it right to amputate them.

VI. *Remarks on the ufe of belladona, by* Mr. Evers.—The firft cafe in which this medicine was given by the author was in a fchirrous affection of the uterus. The patient, we are told, had been eight times pregnant, and was delivered each time time of a dead child. In her ninth pregnancy Mr. Evers was consulted. She was delivered as before of a dead child, and the placenta, upon examination, was found to be attached to a kind of sac, the surface of which was rough like the head of a cauliflower. The patient was directed to take five grains of the powder of belladona, and the same quantity of rhubarb every morning for four weeks, interposing the use of a purgative occasionally. The author informs us that soon after this, his patient became pregnant again, and was delivered of a live and healthy child.—He relates the case of another woman who, after having been delivered of two dead children, complained of dyspnœa, nausea, oppression at the stomach, and continual pain in the left hypochondrium: to this patient also he prescribed the belladona in the same manner as in the other case, and she was afterwards delivered of a live child.

To these cases Mr. Evers has added two instances of herpes; one of these was apparently venereal, and was cured by the mercurius corrosivus sublimatus; the other seeming to be a local affection of the skin, was removed by blisters which were applied to different parts of the body in succession.

VII. *Of*

VII. *Of a ſtone in the rectum, by* Mr. Schwind. —We have here an account of a woman who for a long time complained of pain in her abdomen, and was ſubject to frequent colics. At length a ſtone of the ſize of an hen's egg was accidentally diſcovered in the rectum by giving a glyſter, and was extracted by means of a pair of forceps.

VIII. *Obſervations on the uſe of belladona, by* the ſame.—In a caſe of a cancerous ulcer of the foot this remedy, we are told, was given without effect. At length two grains of the powder were adminiſtered at once. This ſoon occaſioned the moſt alarming ſymptoms, but they ſpeedily diſappeared. The patient died after taking ſix drachms of the medicine.

IX. *An account of a wound of the thorax, by* Mr. Ollenros.—In this caſe part of the left lobe of the lungs, the pericardium, and even the apex of the heart were wounded by a ſword. The ſymptoms that enſued were a ſonorous reſpiration, intermitting pulſe, and ſpaſms of the thorax and extremities. Notwithſtanding theſe alarming appearances a perfect cure was effected in about five weeks.

X. *Of the efficacy of lime water in internal ulcers, by* the ſame.—Two caſes are related in which this remedy was uſed with ſucceſs. In one one of these the patient, who had a purulent expectoration, hectic fever, colliquative sweats, and œdematous swellings of the scrotum and feet, was cured by drinking about a pint and a half of lime water and milk every morning. In fourteen days the expectoration and fever were much lessened. Our author then prescribed the Peruvian bark along with the lime water, and in about four weeks after this the patient was perfectly recovered. In the other case, an ulceration of the urinary bladder, the same remedy proved equally efficacious. Mr. Ollenros observes that it commonly promotes the secretion by the kidneys, but gradually weakens the stomach, on which account its use ought to be occasionally suspended for two or three days, during which time strengthening remedies should be administered.

XI. *Of a wound of the head, by* the same.—In this case one of the parietal bones was injured, but the fracture, we are told, extended only to the diploë. The patient was deprived of his senses by the blow, but recovered them by repeated blood-letting and glysters, and the use of cold applications to the wound; on the third day he complained of pain at the forepart of his head, and of a spasmodic affection of his eyes.

 These

These ſymptoms however were ſoon removed, part of the parietal bone exfoliated, and the wound healed without difficulty.

XII. *On the effects of cold water in caſes of incarcerated hernia, by* the ſame.—Two caſes are related in which this application was tried with ſucceſs. In the firſt of theſe ſnow was applied to the tumour, and renewed as faſt as it melted. Recourſe was had at the ſame time to the tobacco glyſter, and in about ſixteen hours the hernia was reduced. In the ſecond caſe the patient was troubled with vomitings, ſingultus, and a burning pain in the abdomen. Cold water with ſal ammoniacum, nitre and vinegar, were applied to the ſwelling, and the fumes of tobacco thrown up the inteſtines as in the former caſe. The hernia was reduced in about nine hours.—Mr. Ollenros adds a caſe of *prolapſus ani* of long ſtanding, which, after having reſiſted a variety of remedies, was cured in about twenty-four hours by cold water applied to the part, and thrown up by way of glyſter.

XIII. *Another caſe of a wound of the head, by* the ſame.—In this caſe the temporal bone was depreſſed by a fall. The patient became ſenſeleſs, and his pulſe was imperceptible, but upon laying bare the bone, and opening a branch of the the temporal artery, his refpiration returned. Cloths wet with cold water were applied to the wound, and renewed every half hour. The day following the patient was fenfible. On the fifth day the depreffed bone was elevated, and ten ounces of coagulated blood difcharged. On a more clofe examination it was difcovered that the *arteria meningea media* was wounded. On the twelfth day the cold applications were difcontinued; but were repeated again on the fifteenth, on account of an eryfipelatous eruption on the face, attended with fever, and a deep feated pain of the head. Thefe alarming fymptoms, however, foon went off, and the patient gradually recovered.

XIV. *A cafe of deafnefs and blindnefs produced by metaftafis, by* Mr. Püfchel.—In this patient bleeding, blifters, and other remedies were tried without effect. At length the author had recourfe to the method recommended by Mr. Schmucker in his remarks on amaurofis. Half a grain of emetic tartar was given four times a day, with ten grains of gum ammoniac. The dofe of this medicine was gradually increafed till it excited vomiting. In about twelve days the patient began to perceive light, and in a fortnight after this could diftinguifh objects. At length both his ſight and hearing were re-eſtabliſhed.—In another caſe of the ſame kind a ſimilar mode of treatment was adopted, and with equal ſucceſs.

[ *To be continued.* ]

